# STAT3-induced up-regulation of lncRNA NEAT1 as a ceRNA facilitates abdominal aortic aneurysm formation by elevating TULP3

**DOI:** 10.1042/BSR20193299

**Published:** 2020-01-14

**Authors:** Bing Cai, Baihui Yang, Dong Huang, Di Wang, Jun Tian, Feiyun Chen, Xi Wang

**Affiliations:** Cardiovascular Surgery, Second Affiliated Hospital of Kunming Medical University, Kunming, Yunnan, 650101, China

**Keywords:** AAA, miR-4688, NEAT1, STAT3, TULP3

## Abstract

Long noncoding RNAs (lncRNAs) were viewed as crucial participants in the pathogenesis of abdominal aortic aneurysm (AAA). LncRNA NEAT1 was recognized as an oncogenic gene in various diseases. However, its function and mechanism in AAA were not precisely documented. Here, we explored the functional role and molecular mechanism of NEAT1 in AAA. Functionally, the effect of NEAT1 on the proliferation was assessed by CCK-8 and EdU assay, while its impact on the apoptosis was evaluated through caspase-3/9 activity and TUNEL assays. As a result, we found that NEAT1 knockdown enhanced the proliferation and impaired the apoptosis of vascular smooth muscle cells (VSMCs). Reversely, overexpressed NEAT1 exerted anti-proliferation and pro-apoptosis effects in VSMCs. Mechanically, we found that STAT3 acted as a transcription factor and contributed to NEAT1 transcription by ChIP and luciferase reporter assays. In addition, NEAT1 was confirmed as a sponge of miR-4688 and thereby increase the expression of TULP3 in VSMCs via RIP assay and RNA pull-down assay. Rescue experiments indicted that TULP3 overexpressing countervailed the impact of NEAT1 depletion on AAA biological processes. Conclusively, lncRNA NEAT1 induced by STAT3 was identified as a ceRNA and facilitated AAA formation by targeting miR-4688/TULP3 axis.

## Introduction

Abdominal aortic aneurysm (AAA) is identified as one of the most challenging cardiovascular disease and defined as a local dilation of abdominal aorta with a diameter >3.0 cm. Besides, AAA is a relatively common disease, and rupture of AAA is highly possible to cause a life-threatening condition [1]. The mortality rate of ruptured AAA is >80% and ruptured AAA ranks the 13th major cause of death in U.S.A. What’s more, AAA is pathologically featured with inflammatory cell infiltration, extracellular matrix remodeling, integrity decrease of the arterial wall and vascular smooth muscle cells (VSMCs) apoptosis [2]. Although the understanding in AAA physiopathology has been improved, the potential molecular mechanism underling AAA development is still elusive.

Noncoding RNAs (ncRNAs) are a group of RNAs limited to code proteins and are classified as long ncRNAs (lncRNAs) and microRNAs (miRNAs) [3]. Existing evidence corroborated that lncRNAs possess comprehensive biological functions and its dysregulation are correlated with the occurrence of dieases, including AAA [4,5]. For instance, lncRNA DANCR facilitates nasopharyngeal carcinoma metastasis through stabilizing HIF-1α and interacting with NF90/NF45 complex [6]. ZEB1-induced HCCL5 is up-regulated in hepatocellular carcinoma and promotes cancer malignancy [7]. Overexpressed lncRNA HOTAIRM1 contributes to glioblastoma tumor growth and invasion [8]. MiRNAs are also identified as a regulator in a wide range of biological processes and are reported to interact with lncRNA, forming a regulatory network at post-transcriptional level [9,10]. LncRNA nuclear paraspeckle assembly transcript 1 (NEAT1) was reported as an oncogene in breast cancer [11], osteosarcoma [12] and hepatocellular carcinoma [13]. In addition, NEAT1 also played an important regulatory role in some diseases, such as acute kidney injury [14], neuropathic pain [15] and liver fibrosis [16]. Previous study has shown that NEAT1 was overexpressed in AAA [17]; nevertheless, the function and mechanism of NEAT1 in AAA remain obscure.

In the present study, we explored NEAT1 functional role and underlying mechanism in AAA. The results manifested that NEAT1 was induced by STAT3 and promoted AAA formation by sponging miR-4688 and targeting TULP3, providing a novel regulatory axis of STAT3/NEAT1/miR-4688/TULP3 in AAA.

## Materials and methods

### Cell culture

ScienCell Research Laboratories (San Diego, CA, U.S.A.) was the supplier of vascular smooth muscle cells (VSMCs). RPMI-1640 medium (Thermo Scientific, Waltham, U.S.A.) was applied for VSMCs culture, and meanwhile 10% fetal bovine serum and penicillin/streptomycin solution was also needed. The humidified incubator contained 5% CO_2_ and maintained at 37°C.

### VSMCs transfection

Overexpression vectors (pcDNA3.1/NEAT1, pcDNA3.1/STAT3), miR-4688 mimics/inhibitor, miR-146a-5p mimics, miR-204 mimics, miR-98-5p mimics and NC mimics/inhibitor were obtained from RiboBio (RiboBio, Guangzhou, China). Short hairpin RNAs targeting NEAT1 and STAT3 (sh-NEAT1#1/2/3, sh-STAT3#1/2/3) and control sh-NC were supplied by Genepharma (Shanghai, China). VSMCs transfections were achieved by Lipofectamine 2000 (Invitrogen, Carlsbad, CA, U.S.A.).

### Quantitative real-time polymerase chain reaction (qRT-PCR)

TRIzol (Pufei, Shanghai, China) was first used for isolating total RNA from VSMCs. Synthesis of first strand cDNA was completed with the instructions of PrimerScript RT Reagent Kit (TaKaRa, Kyoto, Japan). LightCycler 480 SYBR Green I Master (Roche, Basel, Switzerland) was adopted for performing qRT-PCR. Normalization of target gene expression was conducted by GAPDH expression and results of qRT-PCR underwent the analysis of 2^−ΔΔCt^ method. The utilized premiers are listed in Table 1.

**Table 1:** Primers for qRT-PCR

Genes	Forward sequence (5′–3′)	Reverse sequence (5′–3′)
NEAT1	TTC TCT AGT GTT CCT CAT GGC	TCC TGC AAT GCT AGG ACT C
STAT3	CGC ACT TTA GAT TCA TTG ATG C	AGG TGA GGG ACT CAA ACT G
TULP3	AGC CAA CTA CCT TAT CTC CA	AGG TTG GAT CTA AGC TTG C
miR-770-5p	GUA CCA CGU GUC AGG GC	CTC TAC AGC TAT ATT GCC AGC CAC
miR-4739	GGG AGG AGA GGC GGA G	CTC TAC AGC TAT ATT GCC AGC CAC
miR-4688	GGC AGC AGA GGA CCU GG	CTC TAC AGC TAT ATT GCC AGC CAC
GAPDH	GAA GGT GAA GGT CGG AGT C	GAA GAT GGT GAT GGG ATT TC
U6	ATT GGA ACG ATA CAG AGA AGA TT	GGA ACG CTT CAC GAA TTT G

### Western blot assay

Briefly, VSMCs were washed by PBS twice followed by the addition of cell lysis buffer (ABGENT, Suzhou, Jiangsu, China). Then, collection of VSMCs lysates was performed and a BCA assay kit (MaiRuiBo, Chaoyang, Beijing, China) confirmed the concentrations of proteins. SDS-PAGE (10%) was for separating VSMCs lysates, and consequent proteins were transferred on PVDF membranes, followed by 5% BSA (in TBST buffer) blockade for 1 h. Afterwards, primary antibody against STAT3 (CST, Pudong, Shanghai, China), TULP3 (abcam) or GAPDH (Protein Tech Group, Wuhan, Hubei, China) was used for probing the membranes for 12 h at 4°C. Sequentially, matched secondary antibodies were employed for incubation. A Super ECL assay kit (YRBIO, Changsha, Hunan, China) was for protein band visualization.

### Cell counting kit-8 (CCK-8) assay

To begin with, transfected VSMCs were seeded in 96-well plates (1 × 10^4^ cells/well). Later, 10 µl of CCK-8 solution (Dojindo, Tokyo, Japan) was added for additional incubation of 1 h. Then, a microplate reader (Thermo Fisher Scientific, Waltham, MA, U.S.A.) was employed to detect absorbance at 450 nm at 0, 24, 48 or 72, 96 h.

### 5-ethynyl-2-deoxyuridine (EdU) assay

After transfection, VSMCs were planted into 96-well plates. Subsequently, EdU (50 μM; 5-Ethynyl-2′-deoxyuridine; Beyotime, Haimen, Jiangsu, China) was employed for treating the VSMCs at 37°C for approximately 2 h with 5% CO_2_. VSMCs following formaldehyde (4%), fixation and 1× Click Reaction Buffer (Beyotime, Haimen, Jiangsu, China) were processed for appropriate 30 min without sunlight, followed by DAPI staining. Results of EdU were observed and analyzed under an EVOS M5000 microscope (Thermo Fisher Scientific, Pudong, Shanghai, China).

The ratio of the number of EdU-positive cells (red cells) to the total number of DAPI-positive cells (blue cells) was considered as the EdU incorporation rate.

### Terminal deoxynucleotidyl transferase dUTP nick-end labeling (TUNEL) analysis

Strictly following the manufacturer’s specifications, TUNEL assays applying Apoptosis Detection Kit (Ribobio, China) were carried out on VSMCs. To begin with, 4% paraformaldehyde in PBS was applied to fix each cell line for a quarter at room temperature. Afterwards, PBS washed the cells. And 3% FBS was utilized to block the cells for 1 h at room temperature. Based on the instructions of the manufacturer, the samples were cultured with TUNEL reagent. After being stained with DAPI for 10 min and rinsed with PBS, cells were observed under a fluorescence microscope. TUNEL-positive VSMCs were determined in a randomly selected field. According to total nuclei number and green nucleus, the calculation of TUNEL index was conducted.

### Caspase-3/9 activity assay

Under the detection of a caspase-3 and -9 activity assay kit (Beyotime, Haimen, Jiangsu, China), VSMCs apoptosis was monitored. In short, VSMCs lysates undergoing different treatments were gathered through adding lysis buffer (ABGENT, Suzhou, Jiangsu, China). Thereafter, VSMCs lysates were supplemented with 2 mM of Asp-Glu-Val-Asp (DEAD, for caspase–3) and 2 mM Leu-Glu-His-Asp (LEHD, for caspase-9) labeled with p–nitroaniline (pNA) at 37°C for 2 h in the dark. Absorbance at 405 nm was measured by Labserv K3 microplate reader (WoYuan, Hongkou, Shanghai, China).

### Chromatin immunoprecipitation (ChIP)

Based on the recommendations of the EZ-ChIPTM kit (Millipore, Billerica, MA), a chromatin immunoprecipitation (ChIP) assay was performed to investigate the binding of STAT3 to NEAT1 promoter. The following were the antibodies utilized for immune-precipitating crosslinked protein–DNA complexes: anti-IgG (Millipore) and anti-STAT3 (abcam). The EZ-ChIPTM kit was employed to purify DNA samples. qRT-PCR analysis was designed for the purified immunoprecipitated DNA.

### RNA immunoprecipitation (RIP)

Briefly, a Magna RIP™ RNA-Binding Protein Immunoprecipitation Kit (Millipore, Billerica, MA) was utilized for RIP experiment. Cell lysis of VSMCs was incubated with RIP buffer containing magnetic beads conjugating anti-Ago2 antibody (Cell Signaling Technology, Beverly, MA), and these co-precipitated RNAs by anti-Ago2 antibody were isolated, purified and analyzed by qRT-PCR.

### RNA pull-down assay

NEAT1 biotin probe and NEAT1 control probe purchased from GenePharma were developed into probe-coated beads by cultivating with M-280 Streptavidin magnetic beads (Invitrogen). The miR-4688-WT, miR-4688-Mut and NC were biotin labeled into Bio-miR-4688-WT, Bio-miR-4688-Mut and Bio-NC. Then, cells were lysed and cultivated with probe-coated beads at 4°C. Being cleaned, RNA complexes bound to the beads were eluted and separated. Finally, RNA complexes were detected by qRT-PCR.

### Luciferase reporter assay

The wild-type or mutant type of STAT3-binding sites to NEAT1 promoter was separately subcloned into pGL3 luciferase reporter vector (Promega, Madison, U.S.A.). A fragment from the binding site of NEAT1 or TULP3 was amplified by PCR, and the PCR products were subcloned into pmirGLO vector (Promega) for the generation of NEAT1-WT/TULP3-WT luciferase reporters. To construct the corresponding mutant vectors as control, seed region of miR-4688 binding sites was mutated for the generation of NEAT1-MUT/TULP3-MUT. Besides, cells in 96-well plates were co-transfected with above vectors and indicated plasmids for 48 h. Then, a dual Luciferase Assay kit (Promega, Dongcheng, Beijing, China) was for assaying the luciferase activity.

### Subcellular fractionation assay

PARIS kit (Life Technologies, Carlsbad, CA, U.S.A.) was utilized for separating VSMCs nuclear and cytoplasmic fractions. VSMCs were rinsed with pre-chilled PBS twice, and lysed in 0.1% NP40 mixed with 10 mM Ribonucleoside Vanadyl Complex (New England BioLabs, MA, U.S.A.) and protease inhibitor cocktail (Roche, Basel, Switzerland). After being centrifuged, the supernatant was collected. And PBS was utilized to wash the remaining lysate for five times, followed by centrifugation. Cytoplasmic RNAs extraction and nuclear RNAs isolation were obtained, followed by qPCR assay evaluation. U6 and GAPDH represented nuclear and cytoplasmic controls.

### Fluorescence *in situ* hybridization (FISH) assay

FISH assay was carried out as previously described [18]. First of all, 4% formaldehyde was added to incubate with VSMCs for 15 min and then PBS was used to wash them. Fixed VSMCs was then treated with pepsin and ethanol. Consequently, the FISH probes NEAT1 (Ribobio) was employed for mixing the dried VSMCs for 2 min in a hybridization buffer at 80°C. After dehydration, the slides were counterstained using DAPI and confocal microscope (Leica) captured the images.

### Statistical analysis

Data from the analysis of SPSS version 17.0 software (International Business Machines Corporation (IBM), Armonk, NY) are expressed as mean ± standard deviation. Three repeated experiments were needed in the work. Differences of significance were determined with the methods of Student’s *t* test (two-sides) or one‐way ANOVA. *P* < 0.05 was considered as statistical significance.

## Results

### NEAT1 induced apoptosis and inhibited proliferation of VSMCs

**Figure 1:**
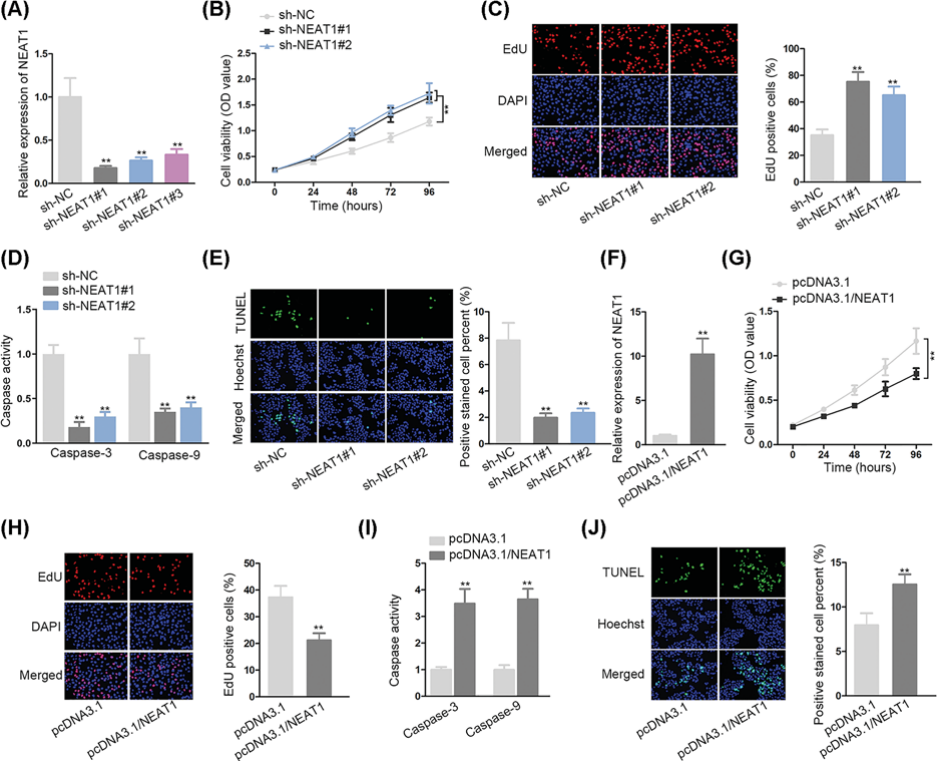
NEAT1 induced apoptosis and inhibited proliferation of VSMCs (**A**) NEAT1 was successfully depleted in VSMCs, as shown in qRT-PCR. (**B,C**) NEAT1 depletion on VSMCs viability and proliferation was assessed by CCK-8 assay and EdU assay. (**D,E**) VSMCs apoptosis after NEAT1 inhibition was assayed by caspase-3/9 activity and TUNEL. (**F**) qRT-PCR tested NEAT1 expression following the transfection of pcDNA3.1/NEAT1. (**G,H**) VSMCs proliferation was determined by CCK-8 and EdU upon NEAT1 overexpression. (**I,J**) VSMCs apoptosis in pcDNA3.1/NEAT1 transfected cells was measured by caspase-3/9 activity and TUNEL; **P < 0.01.

As an oncogenic lncRNA in several cancers, NEAT1 was also revealed to be overexpressed in AAA [17]. Therefore, we aimed to explore the impact of NEAT1 on AAA development. Accordingly, VSMCs were transfected with sh-NEAT1 and results manifested that NEAT1 expression was stably silenced by sh-NEAT1 transfection (Figure 1A). Owing to the optimal transfection efficiency, sh-NEAT1#1 and sh-NEAT1#2 were used for the subsequent experiments. CCK-8 and EdU assays pointed that VSMCs proliferation was elevated by silenced NEAT1 (Figure 1B,C). Subsequently, VSMCs apoptosis was testified by Caspase-3/9 activity and TUNEL assays. Results revealed that knockdown of NEAT1 efficiently hindered VSMC apoptosis (Figure 1D,E). Meanwhile, we stably augmented NEAT1 expression through the transfection of pcDNA3.1/NEAT1 plasmids. qRT-PCR confirmed and quantified its up-regulated level (Figure 1F). As demonstrated, VSMCs proliferation was reduced after NEAT1 was overexpressed (Figure 1G,H). Conversely, cell apoptosis assays elucidated that NEAT1 knockdown contributed to the increased apoptotic rates of VSMCs (Figure 1I,J). Overall, NEAT1 was a contributor in AAA by promoting apoptosis and impeding proliferation of VSMCs.

### STAT3 induced NEAT1 transcription in VSMCs

**Figure 2:**
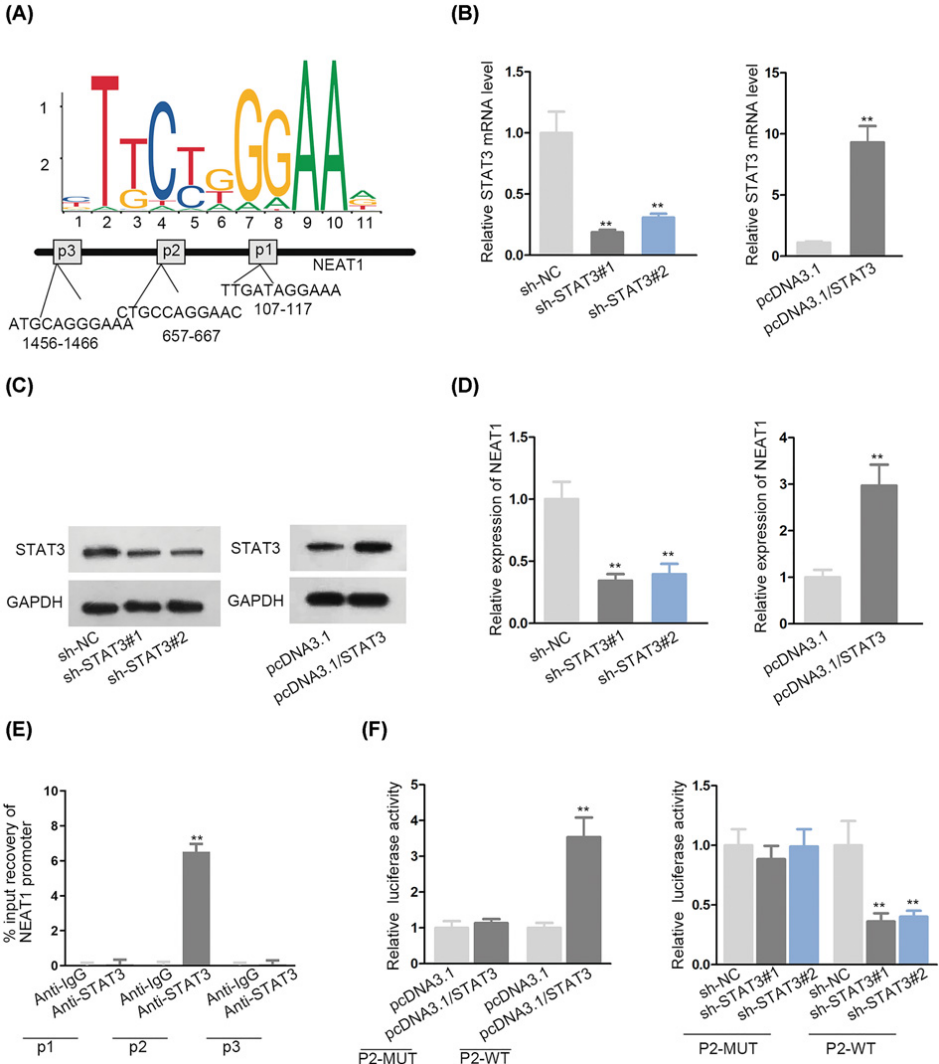
STAT3 induced NEAT1 transcription in VSMCs (**A**) The binding motif of STAT3 to NEAT1 promoter and its response elements in NEAT1 promoter were shown. (**B,C**) Transfection efficiency of sh-STAT3 and pcDNA3.1/STAT3 was validated via qRT-PCR and Western blot assays. (**D**) qRT-PCR was applied to examine the impacts of STAT3 on NEAT1 expression. (**E**) ChIP assay investigated the binding of STAT3 to NEAT1 promoter. (**F**) Luciferase reporter assay interrogated the interaction between STAT3 and NEAT1 promoter; **P < 0.01.

The factor involved in NEAT1 up-regulation in AAA was unclear. Previous studies showed that STAT3 was overexpressed in AAA. Meanwhile, as a potent transcriptional factor, STAT3 could result in the up-regulation of lncRNAs. Based on the results of UCSC (http://genome.ucsc.edu/), STAT3 was predicted as a transcriptional factor of NEAT1. What’s more, the binding motif of STAT3 was presented and top three binding sites to NEAT1 promoter region (binding score>9; p1 site: -107-117, TTGATAGGAAA; p2 site: -657-667, CTGCCAGGAAC; p3 site: -1456-1466, ATGCAGGGAAA) were predicted by JASPAR (http://jaspar.genereg.net/) (Figure 2A). To investigate the impact of STAT3 on NEAT1, STAT3 expression was separately knocked down and overexpressed in VSMCs by transfecting sh-STAT3 and pcDNA3.1/STAT3 (Figure 2B). The transfection efficiency was further confirmed by Western blot assay (Figure 2C). Results of qRT-PCR analysis revealed that NEAT1 expression was decreased upon STAT3 silencing and increased by overexpressed STAT3, indicating the positive regulation of STAT3 on NEAT1 (Figure 2D). Subsequently, we performed ChIP assay to verify the binding sites between STAT3 and NEAT1 promoter. The results uncovered that STAT3 bound to p2 of NEAT1 promoter region (Figure 2E). In subsequence, p2 was mutated for the following luciferase reporter assay and RNA pull-down assay. The results of luciferase reporter assay illuminated that STAT3 overexpression increased the luciferase activity of p2-WT reporter and down-regulation of STAT3 led to decreased luciferase activity of p2-WT reporter, but the effects were invalid when p2 were mutated, implying that STAT3 could bind to NEAT1 promoter at site 2 (Figure 2F). RNA pull-down assay further testified the interaction between STAT3 and NEAT1 promoter (Supplementary Figure S1A). To be concluded, STAT3 was a transcription activator at NEAT1 promoter.

### NEAT1 positively regulated TULP3 through sponging miR-4688 in VSMCs

**Figure 3:**
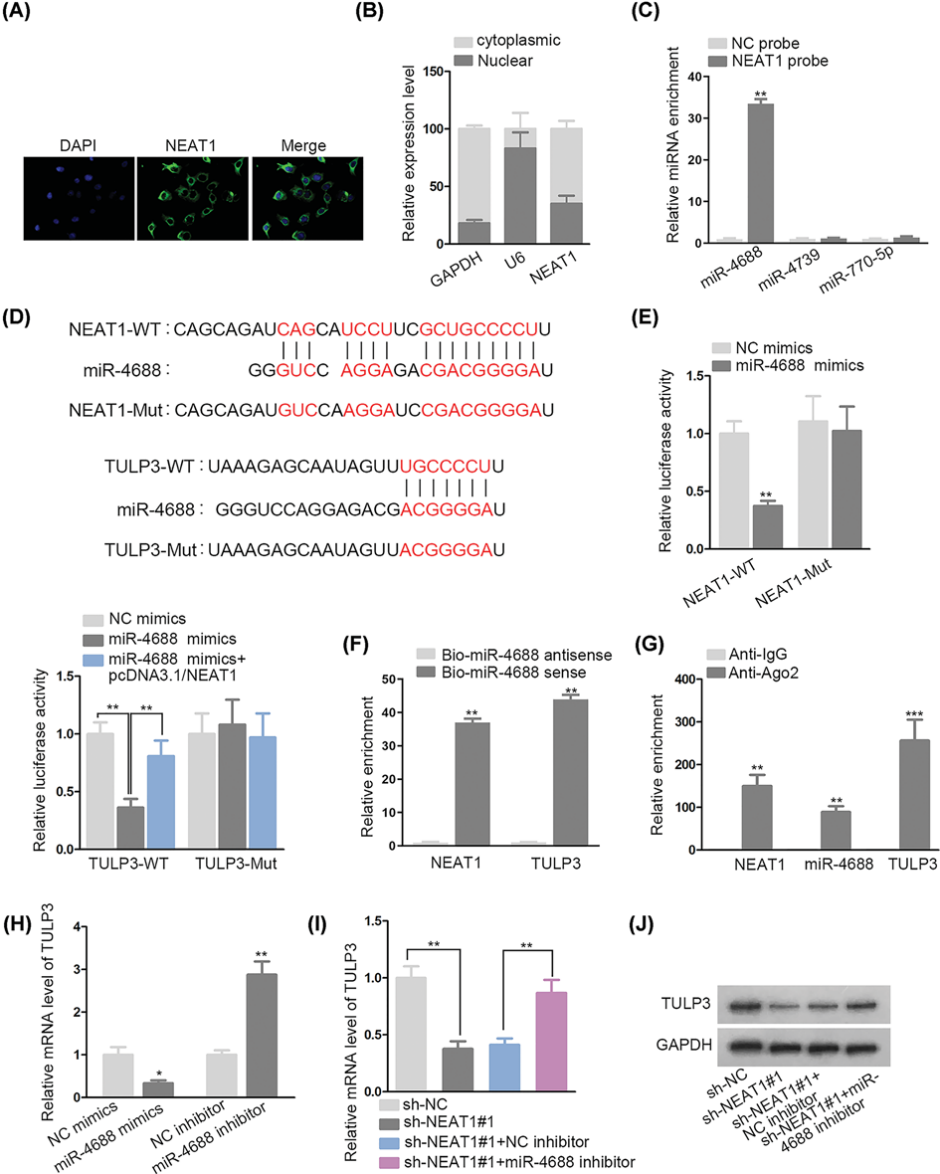
NEAT1 positively regulated TULP3 through sponging miR-4688 in VSMCs (**A,B**) FISH and subcellular fractionation analysis verified the location of NEAT1 in VSMCs. (**C**) NEAT1 pull-down was to monitor the interaction between NEAT1 and three miRNAs predicted by RegRNA 2.0. (**D**) RegRNA 2.0 and miRDB provided the binding sites of miR-4688 on NEAT1 and TULP3. (**E**) Luciferase reporter assay explored the competing binding between miR-4688 and NEAT1 or TULP3. (**F**) RNA pull-down uncovered the association between miR-4688 and NEAT1 or TULP3. (**G**) RIP assay evidenced the co-existence of NEAT1, miR-4688 and TULP3 in RISC. (**H**) The suppressive effect of miR-4688 on TULP3 was ensured using qRT-PCR. (**I,J**) Effects of NEAT1 and miR-4688 on TULP3 expression were checked by qRT-PCR analyisis and Western blot assay; *P < 0.05, **P < 0.01, ***P < 0.001.

To investigate the regulatory mechanism of NEAT1 in AAA, subcellular localization of NEAT1 was determined through FISH assay and subcellular fractionation assay. As a result, NEAT1 was indicated to be chiefly distributed in the cytoplasm of VSMCs (Figure 3A,B). Former evidence showed that lncRNAs act as competing endogenous RNAs (ceRNAs) to up-regulate mRNAs by sponging miRNAs in the cytoplasm [19]. To certify this hypothesis, RegRNA 2.0 (an online tool) was employed and three miRNAs (miR-4688, miR-4739 and miR-770-5p) were predicted to interact with NEAT1. Among the three candidates, miR-4688 was discovered to be significantly enriched in NEAT1 biotin-probe by RNA pull-down assay (Figure 3C). Besides, functional assays implied that miR-4688 overexpression could promote cell proliferation (Supplementary Figure S1B,C) and inhibit cell apoptosis (Supplementary Figure S1D,E). However, AAA cellular process was not affected by the transfection of miR-4739 mimics or miR-770-5p mimics (Supplementary Figure S1F–M). To continue, we decided to uncover the mRNAs that could be targeted by miR-4688. Using miRDB software, TULP3 presented the highest affinity to miR-4688. Furthermore, the binding sequence of miR-4688 on NEAT1 or TULP3 was predicted and the mutant sites of NEAT1 and TULP3 were constructed (Figure 3D). It was widely known that luciferase reporter assay could be used to confirm the interaction between miRNAs and lncRNAs, including NEAT1 [20]. Many miRNAs, such as miR-146a-5p, miR-204 and miR-98-5p, were reported to be sponged by NEAT1 in other diseases. However, they couldn’t influence the luciferase activity of NEAT1-WT/Mut reporter via luciferase reporter assay, suggesting that they couldn’t interact with NEAT1 in AAA (Supplementary Figure S2A–C). Interestingly, the luciferase activity of NEAT1-WT could be notably hampered by miR-4688 mimics, while no changes were viewed in NEAT1-Mut reporter. Furthermore, miR-4688 promotion also decreased TULP3-WT luciferase activity, which could be reversed by NEAT1 up-regulation, revealing that NEAT1 could sponge miR-4688 to upregulate TULP3 (Figure 3E). Moreover, RNA pull-down assay reflected that miR-4688 was closely interacted with NEAT1 and TULP3 (Figure 3F and Supplementary Figure S2D). As reported, miRNAs could exhibit the function of gene silencing by RNA-induced silencing complex (RISC) in which Ago2 is the core component [20]. To determine whether NEAT1, miR-4688 and TULP3 co-exist in the same RISC, we conducted RIP assay and discovered higher enrichment of NEAT1, miR-4688 and TULP3 in Ago2 immunoprecipitates compared with control IgG immunoprecipitates (Figure 3G). In addition, qRT-PCR implied that miR-4688 exerted suppressive function on TULP3 expression (Figure 3H). More importantly, we found that TULP3 mRNA and protein expressions declined by NEAT1 depletion could be abrogated by miR-4688 suppression (Figure 3I,J). Collectively, NEAT1 positively regulated TULP3 through sponging miR-4688 in AAA.

### NEAT1 regulated VSMCs proliferation and apoptosis by elevating TULP3 expression

**Figure 4:**
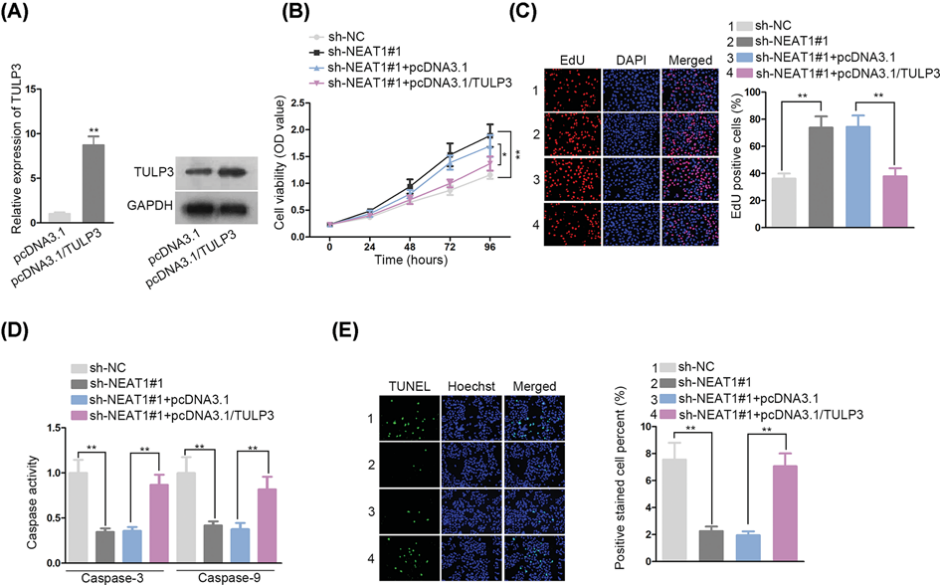
NEAT1 regulated VSMCs proliferation and apoptosis by elevating TULP3 expression (**A**) qRT-PCR and Western blot were executed for detecting the transfection efficacy of pcDNA3.1/TULP3 in VSMCs. (**B,C**) VSMCs proliferation in response to the indicated plasmids was examined by CCK-8 and EdU. (**D,E**) Effects of pcDNA3.1/TULP3 on sh-NEAT1-mediated VSMCs apoptosis were investigated by caspase-3/9 activity and TUENL assay; *P < 0.05, **P < 0.01.

To further confirm the effect of NEAT1/miR-4688/TULP3 axis in AAA, rescue experiments were implemented. TULP3 mRNA and protein levels were overexpressed in VSMCs following pcDNA3.1/TULP3 transfection (Figure 4A). First, CCK-8 and EdU assays were carried out to assess the proliferation. As a result, the impact of down-regulated NEAT1 on VSMCs proliferation was attenuated by TULP3 elevation (Figure 4B,C). Later, the apoptosis was evaluated by caspase-3/9 activity assay and TUNEL assay. The results demonstrated that the inhibitory effect of NEAT1 silencing on VSMCs apoptosis was countervailed through the overexpression of TULP3 (Figure 4D,E). These observations validated that NEAT1 regulated VSMCs proliferation and apoptosis by elevating TULP3 expression.

## Discussion

AAA is considered as a common and challenging disease and ruptured AAA is accompanied with a high risk of death [1]. Up to now, various lncRNAs were reported to regulate the development of AAA. For example, lncRNA H19 promotes AAA formation and vascular inflammation via regulating IL-6 expression [21]. In addition, LINC00265 was also recognized to boost AAA inflammation by sequestering let-7a [22]. As one well-identified lncRNA, NEAT1 was elucidated as a facilitative factor in the carcinogenesis and aggravation, and a reliable prognostic marker in human carcinomas. LncRNA NEAT1 down-regulation was found to restrain the development of gastric cancer and myeloma [23,24]. Importantly, NEAT1 was elevated in AAA through the previous research [17]. Therefore, its function in AAA formation was attracted considerable attention. Here, it was unveiled that NEAT1 inhibition led to accelerative proliferation and reductive apoptosis of VSMCs, while NEAT1 overexpression hindered VSMC apoptosis and exacerbated apoptosis. What’s more, we dissected the mechanism responsible for NEAT1 overexpression in AAA. It presented that STAT3, another overexpressed gene in AAA [25], served as a transcriptional factor for NEAT1 and thereby resulted in its transcription activation. These results revealed that NEAT1 was a contributor in AAA and its expression was induced by STAT3.

Based on collective studies, lncRNAs were validated to cross-talk with specific protein-coding genes in miRNA-dependent form during the formation and deterioration of human carcinomas [26,27]. For instance, LncRNA MEG3 functions as a ceRNA to suppress gastric cancer progression through sequestering oncogenic miR-181s and up-regulating Bcl-2 [28]. LncRNA CASC2 hinders epithelial–mesenchymal transition in hepatocellular carcinoma cells through miR-367/FBXW7 axis [29]. Besides, NEAT1 was also revealed to sponge miRNAs in diseases. Among which, miR-146a-5p, miR-204 and miR-98-5p were randomly selected. As reported, NEAT1 accelerates the accumulation of hepatic lipid in nonalcoholic fatty liver disease via binding to miR-146a-5p to increase ROCK1 expression [30]. NEAT1 promotes autophagy and fortifies cell resistance to sorafenib through sponging miR-204 and modulating ATG3 in hepatocellular carcinoma [31]. NEAT1 promotes HMGA2 expression in prostate cancer by acting as a sponge for miR-98-5p [32]. Nevertheless, our research reflected that NEAT1 could not sponge these miRNAs in AAA. Herein, miR-4688 was identified as the downstream gene of NEAT1. Besides, we unmasked that NEAT1 sponged miR-4688 to protect TULP3 from degradation. Importantly, TULP3 was reported as a prognostic marker in colorectal cancer and pancreatic ductal adenocarcinoma [33]. Additionally, we found that TULP3 elevation could reversed the effect of NEAT1 depletion on VSMCs development in AAA.

Current findings in this paper suggested that NEAT1 could be transcriptionally activated by and regulated AAA cellular process by targeting miR-4688/STAT3 axis, providing a new insight into AAA treatment. However, NEAT1 was also reported to interact with many other miRNAs. For example, NEAT1 was reported to sponge miR-101 in non-small cell lung cancer [34], papillary thyroid carcinoma [35], hepatocellular carcinoma [36] and breast cancer [11]. Besides, NEAT1 sponged miR-124 in neuroblastoma [37], retinoblastoma [38], Alzheimer’s disease [38] and nasopharyngeal carcinoma [39]. Furthermore, NEAT1 sponged miR-129 in esophageal squamous cell carcinoma [40] and hepatoblastoma [41]. NEAT1 also sponged miR-335 in hepatocellular carcinoma [42], acute lymphoblastic leukemia [43], gastric cancer [44] and pancreatic cancer [45]. The interaction between above miRNAs and NEAT1 was not explored in our study, which indicating the limitations in the current study. Nevertheless, the other miRNA–NEAT1 interactions will be explored in the future.

## Supplementary Material

Supplementary Figures S1-S2

## Abbreviations

AAA: abdominal aortic aneurysm
ANOVA: analysis of variance
CCK-8: Cell counting kit-8
ceRNA: competing endogenous RNA
ChIP: chromatin immunoprecipitation
EdU: 5-ethyny1-2-deoxyuridine
FBS: fetal bovine serum
FISH: fluorescence *in situ* hybridization
GAPDH: glyceraldehyde-3-phosphate dehydrogenase
IgG: immunoglobulin G
lncRNA: long noncoding RNA
NC: negative control
NEAT1: nuclear paraspeckle assembly transcript 1
miRNAS: microRNAs
ncRNAs: non-coding RNAs
PVDF: polyvinylidene fluoride
qRT-PCR: quantitative Real time polymerase chain reaction
RIP: RNA immunoprecipitation
RISC: RNA-induced silencing complex
SD: standard deviation
SDS-PAGE: sodium dodecy1 sulfate-polyacrylamide gel electrophoresis
STAT3: signal transducer and activator of transcription 3
TULP3: TUB like protein 3
TUNEL: terminal deoxynucleotidyl transferase dUTP nick-end labeling
VSMC: vascular smooth muscle cell
