# Supplementary material for: STAT3-induced up-regulation of lncRNA NEAT1 as a ceRNA facilitates abdominal aortic aneurysm formation by elevating TULP3
Source: Biosci Rep. 2020 Jan 14;40(1):BSR20193299. doi: 10.1042/BSR20193299 (PMC6960067; doi:10.1042/BSR20193299)
Supplement: Supplementary Figures S1-S2 [file BSR-2019-3299_supp.pdf]

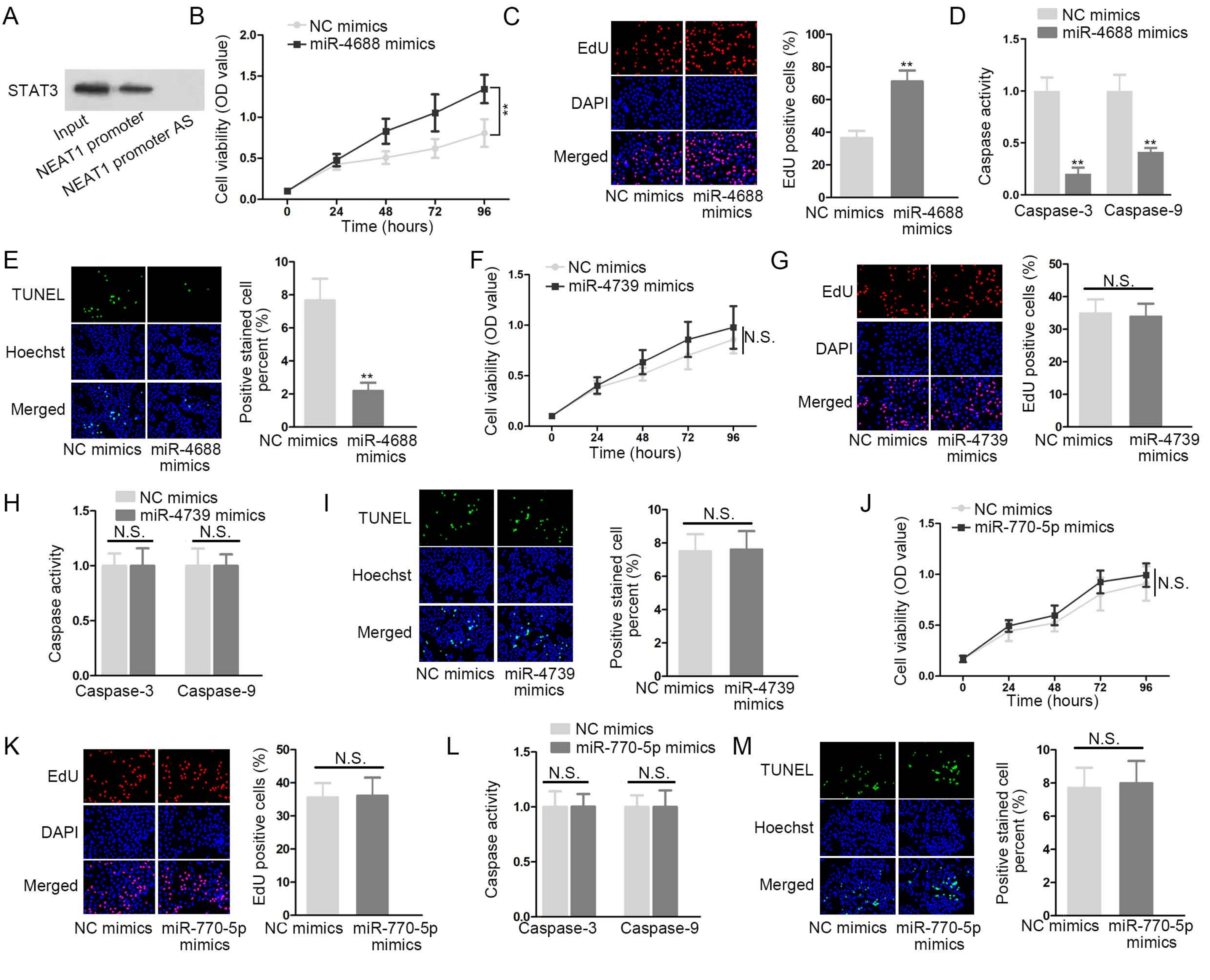

A
STAT3
Input
NEAT1 promoter
NEAT1 promoter AS
B
NC mimics
miR-4688 mimics
Cell viability (OD value)
Time (hours)
**
C
EdU
DAPI
Merged
NC mimics
miR-4688 mimics
EdU positive cells (%)
D
Caspase activity
Caspase-3
Caspase-9
E
TUNEL
Hoechst
Positive stained cell percent (%)
F
miR-4739 mimics
N.S.
G
H
I
J
miR-770-5p mimics
K
L
M

Figure S1.

(A) The binding between STAT3 and NEAT1 promoter was confirmed by RNA pull down assay. (B-C) CCK-8 and EdU assays revealed cell proliferation in miR-4688 mimics-transfected cells. (D-E)Cell apoptosis after miR-4688 overexpression was explored by caspase-3/9 activity and TUNEL assays. (F-G) Cell proliferative ability of miR-4739 mimics-transfected cells was demonstrated byCCK-8 and EdU assays. (H-I)Caspase-3/9 activity and TUNEL assays measured cell apoptotic ability after the transfection of miR-4739 mimics. (J-K)The influence of miR-770-5p overexpression on cell proliferation was examined by CCK-8 and EdU assays. (L-M) Caspase-3/9 activity and TUNEL assays evaluated the effect of miR-770-5p upregulation on cell apoptosis.**P<0.01,N.S. presented no significance.

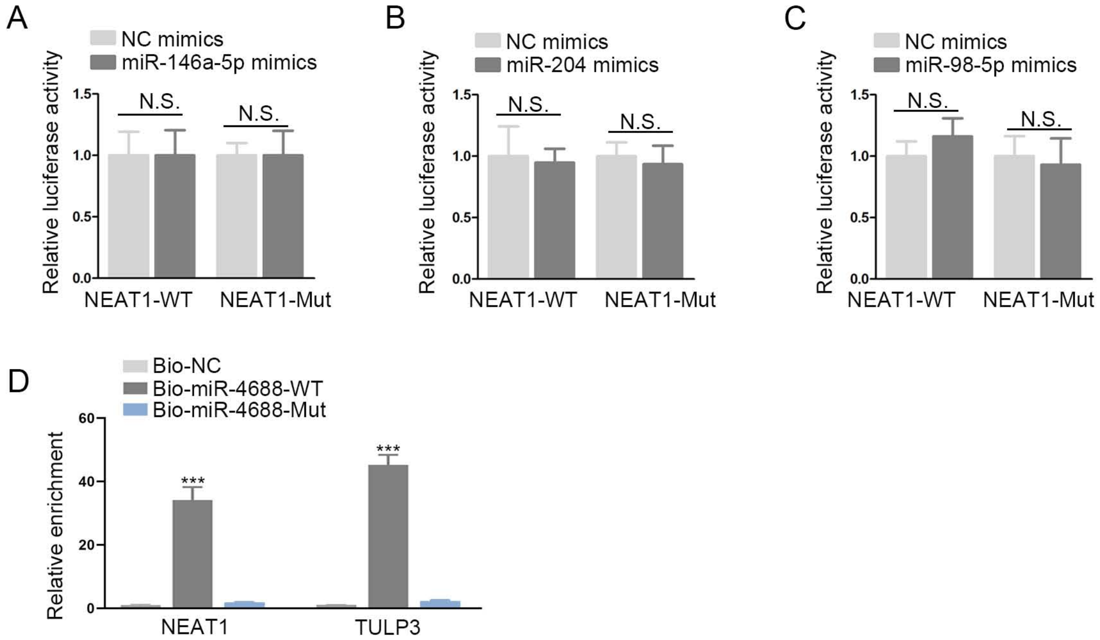
A
NC mimics
miR-146a-5p mimics
Relative luciferase activity
1.5
1.0
0.5
0.0
N.S.
N.S.
NEAT1-WT
NEAT1-Mut
B
NC mimics
miR-204 mimics
Relative luciferase activity
1.5
1.0
0.5
0.0
N.S.
N.S.
NEAT1-WT
NEAT1-Mut
C
NC mimics
miR-98-5p mimics
Relative luciferase activity
1.5
1.0
0.5
0.0
N.S.
N.S.
NEAT1-WT
NEAT1-Mut
D
Bio-NC
Bio-miR-4688-WT
Bio-miR-4688-Mut
Relative enrichment
60
40
20
0
***
***
NEAT1
TULP3

Figure S2.

(A-C)Luciferase reporter assay evaluated the luciferase activity of NEAT1-WT/MUT reporter under miR-146a-5p (miR-204 or miR-98-5p)overexpression.(D)RNA pull down assay further validated the combination of miR-4688 with NEAT1 or TULP3.***P<0.001.N.S. showednosignificance.

Primers for qRT-PCR

Genes	Forward sequence (5′–3′)	Reverse sequence (5′–3′)
NEAT1	TTC TCT AGT GTT CCT CAT GGC	TCC TGC AAT GCT AGG ACT C
STAT3	CGC ACT TTA GAT TCA TTG ATG C	AGG TGA GGG ACT CAA ACT G
TULP3	AGC CAA CTA CCT TAT CTC CA	AGG TTG GAT CTA AGC TTG C
miR-770-5p	GUA CCA CGU GUC AGG GC	CTC TAC AGC TAT ATT GCC AGC CAC
miR-4739	GGG AGG AGA GGC GGA G	CTC TAC AGC TAT ATT GCC AGC CAC
miR-4688	GGC AGC AGA GGA CCU GG	CTC TAC AGC TAT ATT GCC AGC CAC
GAPDH	GAA GGT GAA GGT CGG AGT C	GAA GAT GGT GAT GGG ATT TC
U6	ATT GGA ACG ATA CAG AGA AGA TT	GGA ACG CTT CAC GAA TTT G
